# Contrast-free MRI-first screening in young asymptomatic men: Prostate characteristics and cancer detection rates

**DOI:** 10.1038/s41391-026-01116-5

**Published:** 2026-05-13

**Authors:** Emanuele Messina, Ludovica Laschena, Antonella Borrelli, Francesca Mezzapesa, Sara Lucciola, Luca Giuliani, Paolo Giuliani, Marco Bicchetti, Alessandro Sciarra, Valeria Panebianco

**Affiliations:** 1 Department of Radiological Sciences, Oncology and Pathology, Sapienza University / Policlinico Umberto I, Viale del Policlinico 155, 00161 Rome, Italy; 2 Poliambulatorio Montezemolo, Ente Sanitario Militare del Ministero Della Difesa Presso la Corte dei Conti, 00195 Rome, Italy; 3 Department of Maternal-Infant and Urological Sciences, Sapienza University / Policlinico Umberto I, Viale del Policlinico 155, 00161 Rome, Italy

**Keywords:** Outcomes research, Cancer screening

## Abstract

**Objectives:**

Early-onset prostate cancer is becoming increasingly prevalent, and MRI-first strategies are gaining interest as a potential screening tool. However, MRI characteristics of the prostate in younger men remain underexplored. This study aimed to characterize prostate MRI features in a young, asymptomatic male cohort undergoing contrast-free biparametric MRI for prostate cancer screening, to determine the detection rate of clinically significant prostate cancer, and to identify clinical and imaging predictors of clinically significant disease.

**Methods:**

A total of 659 prostate MRIs were acquired; after excluding men who declined biopsy, 641 participants formed the final cohort. Peripheral-zone signal patterns on T2-weighted imaging (T2WI), diffusion-weighted imaging (DWI), and apparent diffusion coefficient (ADC) maps were assessed by two blinded radiologists. PI-RADS scores were assigned independently. Men with PI-RADS ≥ 4 were offered targeted MRI–TRUS fusion biopsy (PI-RADS 3 in case of PSAD ≥ 0.16). Multivariable logistic regression was performed to identify independent predictors of csPCa.

**Results:**

Median PSA 1.02 ng/mL (IQR 0.58–2.03) and PSA density (PSAD) 0.03 ng/mL/mL (IQR 0.02–0.05). The most common peripheral-zone appearance was heterogeneous T2 hypointensity (74.7%), homogeneous DWI hyperintensity (59.4%), and homogeneous ADC hypointensity (66.1%). PI-RADS distribution was: 0.5% PI-RADS 1, 81.1% PI-RADS 2, 14.2% PI-RADS 3, 3.6% PI-RADS 4, and 0.6% PI-RADS 5. Forty-one men (6.4%) underwent biopsy, yielding 5 ciPCa, 23 csPCa, and 13 negative results, corresponding to csPCa in 3.6% of the entire cohort. In multivariable analysis, PSAD ≥ 0.15 ng/mL/mL, PI-RADS 4–5, and family history were independently associated with csPCa (all *p* < 0.01).

**Conclusions:**

Contrast-free bpMRI effectively characterizes prostate morphology in younger men and identifies csPCa at an early stage. PSAD, PI-RADS category, and family history significantly enhance risk stratification, supporting the integration of bpMRI-based approaches into future MRI-first screening strategies for younger, asymptomatic populations.

## Introduction

Prostate cancer (PCa) is a complex and multifactorial disease that requires early detection to enable prompt treatment [1]. It represents a major global health challenge, affecting approximately one in seven men, with incidence and mortality rates being affected by age, family history, and geographic variability [2]. In recent years, a growing body of epidemiological evidence has highlighted a rising incidence of prostate cancer, becoming the second most common cancer in men, also including patients younger than 60 years [3].

To date, no universally accepted screening strategy for PCa has been established, while recommendations of its risk-based early diagnosis have been proposed. While low-risk individuals typically undergo follow-up without imaging, those at intermediate or high risk are referred for prostate magnetic resonance imaging (MRI) [4, 5]. Although PSA screening has been shown to reduce PCa-specific mortality, it is also associated with the detection of a high proportion of clinically insignificant prostate cancers (ciPCa), leading to potential overdiagnosis and overtreatment [6–8].

Conversely, MRI and PI-RADS score have demonstrated high diagnostic accuracy in PCa detection [9, 10]. Moreover, non-contrast biparametric MRI (bpMRI) has emerged as a promising alternative to multiparametric MRI [11–13] and as a potential screening tool, offering shorter acquisition times, eliminating contrast-related risks, and therefore appearing more suitable for large-scale screening or first-line evaluation, especially in younger and lower-risk patients. Recent trials, including ongoing multicenter studies, have investigated the feasibility of MRI-first strategies, underscoring the potential role of bpMRI in early cancer detection [14–18].

Moreover, prostate MRI frequently uncovers incidental or collateral findings beyond the gland itself, such as pelvic vascular anomalies, bone lesions, rectal abnormalities, or lymphadenopathy, which may carry clinical significance, particularly in younger, asymptomatic individuals undergoing imaging for the first time [19, 20]. Despite this, most existing MRI research has predominantly focused on older men at higher risk for PCa, leaving a gap in our understanding of prostate morphology, function, and incidental findings in younger populations.

Given these considerations, a deeper investigation of prostate MRI characteristics in young, prostate cancer–naïve men could provide valuable baseline information and improve our understanding of both normal and potentially suspicious imaging patterns in this demographic.

Accordingly, the primary objective of this study is to characterize the prostate gland’s imaging features in a young, asymptomatic cohort of men with no prior diagnosis of prostate cancer. As secondary aims, we evaluate the detection rate (DR) of clinically significant prostate cancer (csPCa) using biparametric MRI (bpMRI), assess the relationship between MRI findings and relevant clinical or laboratory parameters, and describe the prevalence and nature of incidental extra-prostatic findings identified during imaging.

## Materials & methods

### Study design

This is a prospective, interventional and observational study including a cohort of male subjects, all active-duty military personnel, who underwent prostate biparametric magnetic resonance imaging (bpMRI) as part of a screening program. The study received formal approval from the Local Ethics Committee; written informed consent was obtained from each participant.

Inclusion criteria were age between 40 and 69 years, availability of complete demographic and clinical data, and absence of prior prostate cancer diagnosis or urological interventions. Patients with incomplete records or a history of prostate malignancy were excluded.

### Magnetic resonance imaging acquisition and reporting

All examinations were conducted on one 1.5-Tesla MRI scanners (MAGNETOM Sola Siemens), using a 32-channel surface phased-array pelvic coil, according to all the technical recommendations of PI-RADS v2.1. The protocol includes high-resolution T2-weighted imaging (T2WI) in the axial and coronal planes and diffusion weighted imaging (DWI) at b-values of 100, 800, 1000, and 2000 s/mm2 with apparent diffusion coefficient (ADC) map reconstruction (based on 100, 800, and 1000 s/mm2) and a large field-of-view DIXON sequence without contrast media injection. Protocol characteristics are detailed on Supplementary Table 1.

Two radiologists, both experienced in prostate MRI interpretation (one with more than 15 years of experience and the other one with 5 years of experience), independently analyzed all examinations. Both readers were blinded to clinical, laboratory, and histopathological data. The radiologists assigned a PI-RADS score to each alteration detectable on the bpMRI, according to PI-RADS v2.1 recommendations [9]. A score of 1 or 2 indicates a very low or low probability of PCa, respectively; a score of 4 or 5 indicates a high or very high probability of PCa, respectively. The defined MRI threshold for setting indication to biopsy was PI-RADS score of ≥4 and PI-RADS 3 when associated with PSA-density ≥0.15 ng/mL/mL [21–25].

The peripheral zone was evaluated by both readers on T2-weighted (T2W) images, diffusion-weighted imaging (DWI) and the apparent diffusion coefficient (ADC) maps, who described it as detailed on Table 1.

**Table 1:** Peripheral-zone signal characteristics on T2-weighted imaging (T2WI), diffusion-weighted imaging (DWI), and apparent diffusion coefficient (ADC) maps.

Sequence	Signal Pattern	n.	% (95% CI)
T2WI	Diffusely hypointense ( > 80% low signal)	96	15.0 (12.4–17.9)
	Heterogeneously hypointense (30–80% low signal)	479	74.7 (71.2–77.9)
	Predominantly hyperintense ( < 30% low signal)	66	10.3 (8.2–12.9)
DWI	Normal	69	10.8 (8.6–13.4)
	Heterogeneously hyperintense	139	21.7 (18.7–25.0)
	Homogeneously hyperintense	381	59.4 (55.6–63.2)
	Heterogeneously hypointense	36	5.6 (4.1–7.7)
	Homogeneously hypointense	16	2.5 (1.5–4.0)
ADC maps	Normal	37	5.8 (4.2–7.9)
	Heterogeneously hyperintense	60	9.4 (7.3–11.9)
	Homogeneously hyperintense	6	0.9 (0.4–2.0)
	Heterogeneously hypointense	114	17.8 (15.0–20.9)
	Homogeneously hypointense	424	66.1 (62.4–69.7)

MR image quality proved to be a critical factor [26, 27]; therefore, examinations with low-quality imaging were excluded from the study. To ensure consistency in this assessment, we applied the Prostate Imaging Quality (PI-QUAL) version 2 criteria [28] and included only MRIs classified with a PI-QUAL score of 2 or 3, therefore of acceptable/optimal quality.

### Prostate targeted biopsy

Every patient advised undergoing biopsy underwent an MR-directed biopsy (MRDB) with a targeted approach, utilizing the TRUS (transrectal ultrasound)-MR image fusion technique. The MRDB procedure was carried out by the same experienced radiologists who acquired the MR images. All the samples were stored and preserved formalin-filled containers and analyzed by a pathologist with 15 years of expertise in genitourinary pathology, who documented the biopsy cores using a homogeneous nomenclature.

Clinically significant prostate cancer (PCa) is defined as an International Society of Urogenital Pathology (ISUP) grading group (GG) ≥ 2.

### Statistical analysis

Descriptive statistics were used to summarize patient characteristics and MRI findings. Categorical variables were compared using the Chi-square test or Fisher’s exact test as appropriate.

Between-group differences for continuous variables were evaluated using non-parametric tests (Mann–Whitney U test for two groups, Kruskal–Wallis test for more than two groups).

Cohen’s κ coefficients were calculated to quantify inter-reader agreement for both PI-RADS scoring and peripheral zone signal classification.

Univariable and multivariable analysis with logistic regression was performed to assess which of the following variables was independently predictive of csPCa: patient age (<50 vs. ≥50), family history (positive vs. negative), comorbidities (positive vs. negative) PSA density value (<0.15 vs. ≥0.15 ng/mL/ mL), and the PI-RADS score (<4 vs. ≥4).

The reference standard was defined using a composite approach appropriate for a single-round screening setting: true-positive cases were confirmed by histopathology, whereas true-negative cases were defined by either a negative biopsy or stable findings during follow-up (nonprogressive MRI appearance and/or non-increasing PSA levels).

All tests were two-tailed, and a *p*-value < 0.05 was considered statistically significant. Statistical analyses were conducted using SPSS version 27 (IBM Corp., Armonk, NY, USA).

## Results

### Patient characteristics

A total of 665 prostate MRIs were initially acquired. Eighteen participants (2.7%; 95% CI: 1.72–4.24%) were excluded for declining to undergo targeted biopsy despite radiological indication, and six men (0.9%; 95% CI: 0.41–1.95%) were excluded due to low-quality imaging (PI-QUAL score = 1). The final study cohort thus consisted of 641 men, only including MRIs classified as PI-QUAL score 2 or 3. The median age was 55 years (IQR 53–59). The median prostate-specific antigen (PSA) value was 1.02 ng/mL (IQR 0.58–2.03), and the median PSA density (PSAD) was 0.03 ng/mL/mL (IQR 0.02–0.05).

### MRI characteristics of the young population

**Fig. 1:**
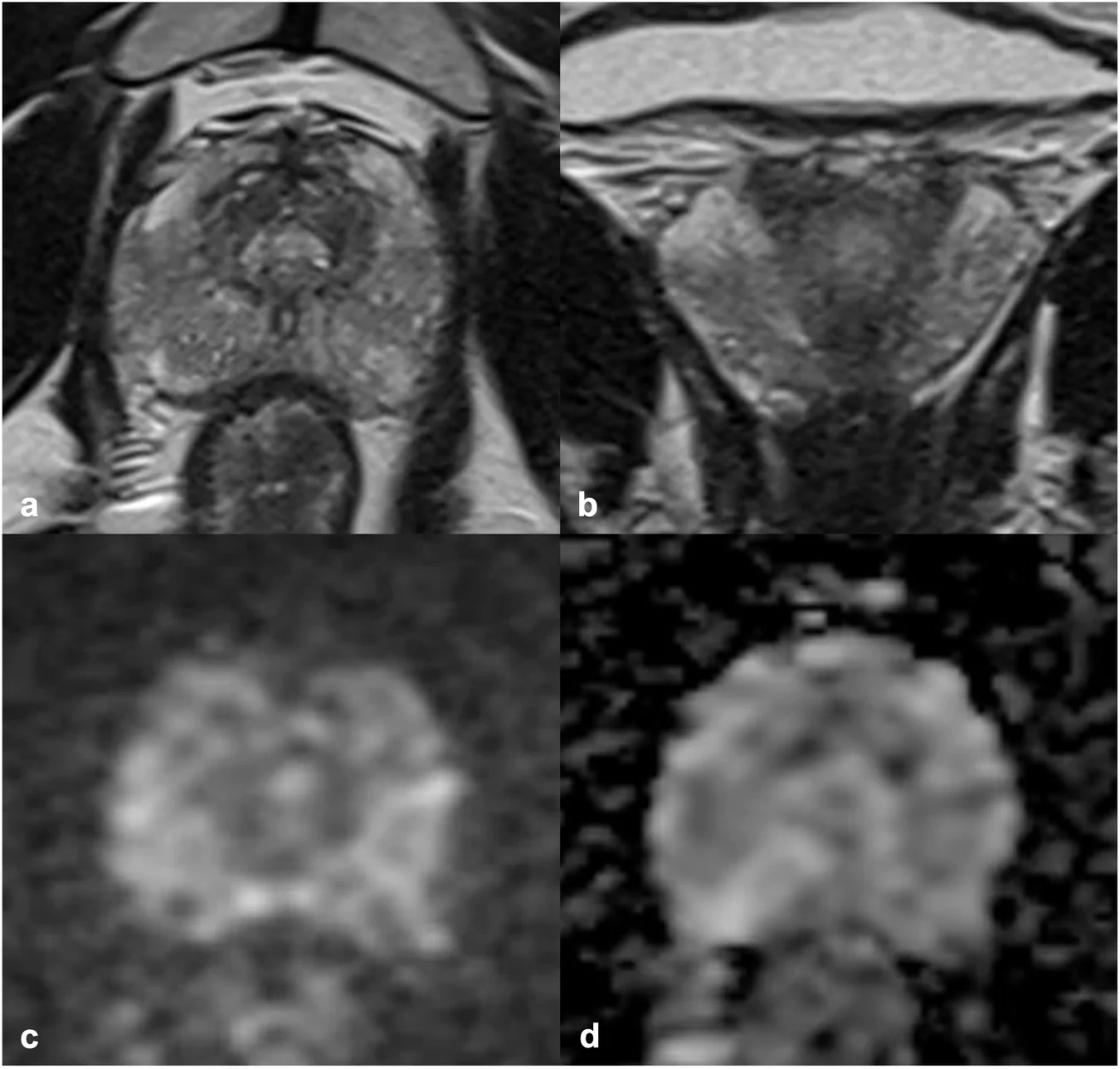
50-year-old man undergoing screening MRI with a PSA value of 0.7 ng/mL and no clinical suspicion of prostate cancer. **a,** **b** axial and coronal T2WI show diffuse heterogeneous hypointensity of the peripheral zone. **c** DWI (b-value = 2000) demonstrates slight homogeneous hyperintensity of the peripheral zone, with corresponding homogeneous diffused hypointensity on the ADC map **d**. No suspicious lesions were identified, and an overall PI-RADS 2 score was assigned; the PSA-Density resulted 0,02 ng/ml^2^. *PSA prostate-specific antigen, T2WI,T2-weighted imaging, DWI diffusion-weighted imaging, ADC apparent diffusion coefficient, PI-RADS Prostate Imaging Reporting and Data System*.

Peripheral-zone signal characteristics on T2WI, DWI, and ADC maps are summarized in Table 1. On T2WI, heterogeneous hypointensity, defined as low signal involving 30–80% of the glandular volume, was the most frequent appearance, observed in 479/641 men (74.7%, 95% CI 71.2–77.9). On DWI, homogeneous hyperintensity was recorded in 381/641 men (59.4%, 95% CI 55.6–63.2), while on ADC maps, homogeneous hypointensity was observed in 424/641 (66.1%, 95% CI 62.4–69.7) (Fig. 1).

Among the 641 men included, 137 (21.4%; 95% CI: 18.4–24.7%) were aged ≥60 years. Of these, 103 (75.2%; 95% CI: 67.3–81.7%) demonstrated an overall typical prostate appearance, characterized by higher T2 signal intensity and absent diffusion restriction, consistent with known age-related imaging features.

Inter-reader agreement for the categorization of peripheral-zone signal on T2WI, DWI, and ADC maps was excellent (κ = 0.85; 95% CI 0.80–0.89).

### PI-RADS distribution

Among the 641 bpMRI examinations analyzed, 3/641 (0.5%, 95% CI 0.2–1.4) were classified as PI-RADS 1, 520/641 (81.1%, 95% CI 78.0–84.1) as PI-RADS 2, 91/641 (14.2%, 95% CI 11.7–17.1) as PI-RADS 3, 23/641 (3.6%, 95% CI 2.4–5.4) as PI-RADS 4, and 4/641 (0.6%, 95% CI 0.2–1.6) as PI-RADS 5 (Table 2).

**Table 2:** Descriptive characteristics of the study population according to histopathological outcome.

Variable	Total Cohort	Negative/Inflammations	ciPCa	csPCa	*p*-value^*^
Sample Size, *n (%)*	641 (100)	613 (95,6)	5 (0,8)	23 (3,6)	**<0,001**
Age (years), *Median (IQR)*	55 (53–59)	55 (53–58)	58 (53–73)	58 (56–67)	0,277
Comorbidities, *n (%)*					
No	594 (92,6)	576 (89,8)	2 (0,3)	16 (2,5)	
Yes	47 (7,4)	37 (5,8)	3 (0,5)	7 (1,1)	< **0,001**
Family history, *n (%)*					
No	580 (90,5)	561 (87,5)	4 (0,6)	15 (2,3)	
Yes	61 (9,5)	52 (8,1)	1 (0,2)	8 (1,2)	< **0,001**
PSA (ng/ml), *Median (IQR)*	1,1 (0,6–2,3)	1,0 (0,6–2,1)	4,3 (1,9–9,5)	3,7 (2,9–6,3)	**<0,001**
Prostate Volume (ml), *Median (IQR)*	34 (26–47)	34 (26–47)	40 (29–60)	37 (34–57)	0,422
PSA Density (ng/ml^2^), *Median (IQR)*	0,03 (0,02–0,06)	0,03 (0,02–0,05)	0,17 (0,04–0,18)	0,13 (0,11–0,16)	**<0,001**
PI-RADS, *n (%)*					
1	3 (0,5)	3 (0,5)	0 (0)	0 (0)	
2	520 (81,1)	520 (81,1)	0 (0)	0 (0)	
3	91 (14,2)	86 (13,4)	3 (0,5)	2 (0,3)	< **0,001**
4	23 (3,6)	3 (0,5)	2 (0,3)	18 (2,8)	
5	4 (0,6)	1 (0,2)	0 (0)	3 (0,5)	

*PSA* prostate-specific antigen, *PI-RADS* Prostate Imaging Reporting and Data System, *ciPCa* clinically insignificant prostate cancer, *csPCa* clinically significant prostate cancer.

Inter-reader agreement for PI-RADS score assignment was substantial, with a Cohen’s κ value of 0.82 (95% CI 0.76–0.86).

### Detection rate

**Fig. 2:**
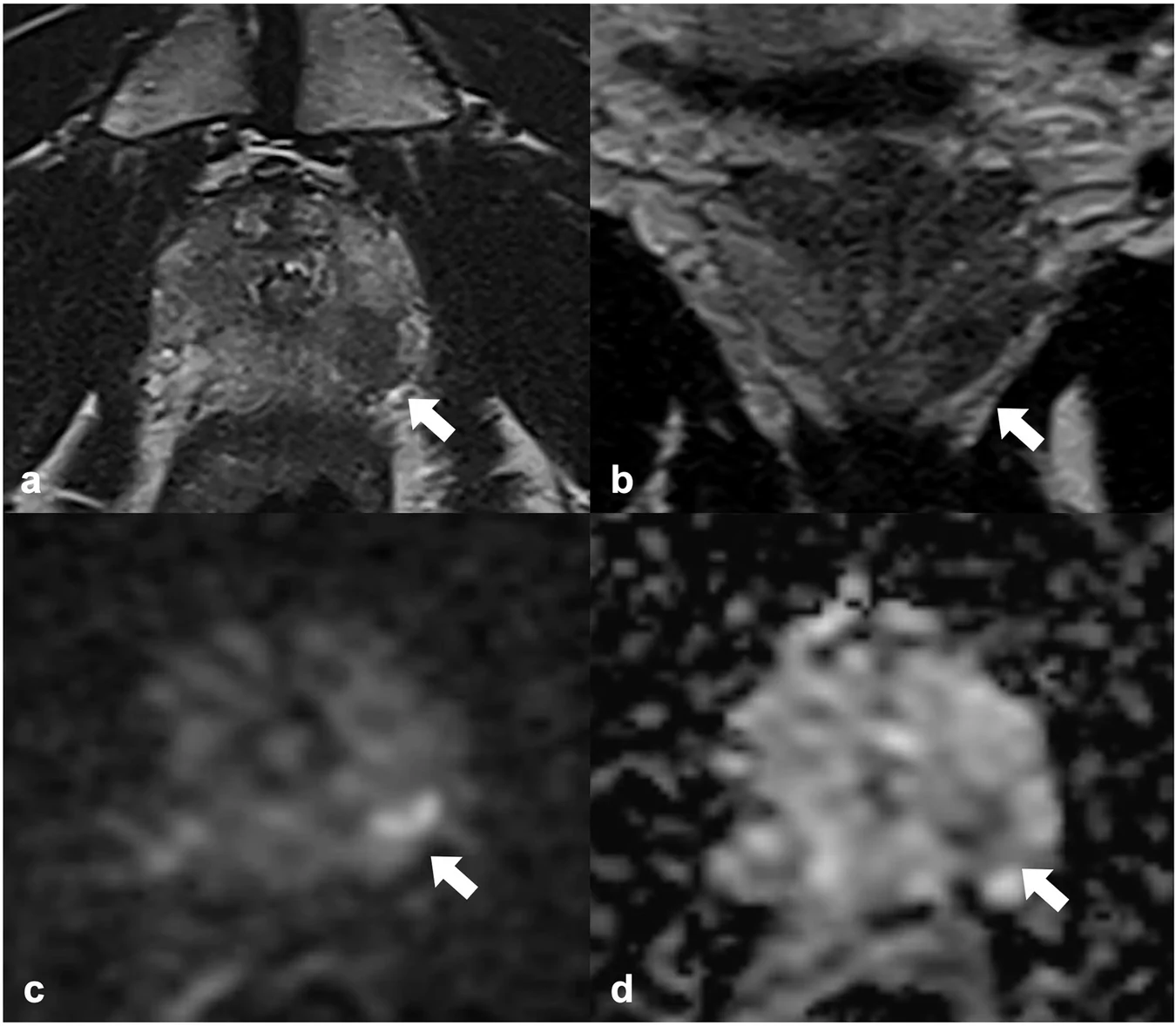
A 54-year-old man with elevated PSA (4.1 ng/mL) and PSA density of 0.17 ng/mL/mL underwent biparametric prostate MRI. **a, b** axial and coronal T2WI reveal a focal hypointense lesion in the left posterolateral peripheral zone at the apex. **c** DWI (b-value 2000) demonstrates corresponding focal hyperintensity, with markedly low signal on the ADC map **d**. The lesion measured 10 mm and it was classified with a PI-RADS 4 score. The lesion was subsequently targeted using MRI–TRUS fusion biopsy, and histopathology confirmed clinically significant prostate cancer, Gleason score 7 (4 + 3). *PSA prostate-specific antigen, T2WI T2-weighted imaging, DWI diffusion-weighted imaging, ADC apparent diffusion coefficient, PI-RADS Prostate Imaging Reporting and Data System, TRUS transrectal ultrasound*.

A total of 41/641 men (6.4%, 95% CI 4.7–8.6) underwent MR-targeted biopsy. Among them, 13/41 (31.7%, 95% CI 17.5–45.9) had negative histopathological results, 5/41 (12.2%, 95% CI 2.2–22.2) were diagnosed with clinically insignificant prostate cancer (ciPCa), and 23/41 (56.1%, 95% CI 40.9–71.3) were found to have csPCa (Fig. 2).

When considering the entire MRI cohort (*n* = 641), this corresponds to 13/641 (2.0%, 95% CI 0.9–3.1) with negative biopsy, 5/641 (0.8%, 95% CI 0.1–1.5) with ciPCa, and 23/641 (3.6%, 95% CI 2.2–5.0) with csPCa.

### Descriptive and multivariate analyses

Comparative demographic and clinical characteristics among subgroups (negative/inflammatory, ciPCa, and csPCa) are reported in Table 2.

In the multivariate logistic regression model (Table 3), PSA density ≥0.15 ng/mL/mL emerged as an independent predictor of csPCa (adjusted OR 0.08, 95% CI 0.05–0.12; *p* < 0.001). Moreover, PI-RADS score (≥4) was also significantly associated with csPCa (adjusted OR 0.78, 95% CI 0.74–0.82; *p* < 0.001). Another factor correlating with csPCa was family history of PCa (adjusted OR 0.04, 95% CI 0.01–0.06; *p* = 0.003). Age and comorbidities were not independently correlated after adjustment for other variables.

**Table 3:** Multivariate logistic regression analysis for predictors of clinically significant prostate cancer (csPCa).

Variables	Evidence of csPCa *n* (%) – 641		P*	Odds Ratio*	P**	Odds Ratio**
	Not	Yes				
Age *(mean)*						
<50	260 (40,6)	2 (0,3)	**0,001**	0,05 (0,02–0,08)	0,715	0,02 (0,01–0,03)
≥50	358 (55,8)	21 (3,3)				
Family History						
NO	565 (88,1)	15 (2,3)	**<0,001**	0,10 (0,06–0,15)	**0,003**	0,04 (0,01–0,06)
YES	53 (8,4)	8 (1,2)				
Comorbidities						
NO	578 (90,2)	16 (2,5)	**<0,001**	0,12 (0,07–0,18)	0,854	0,01 (0,01–0,03)
YES	40 (6,2)	7 (1,1)				
PSA Density *(ng/ml*^*2*^*)*						
<0,15	599 (93,4)	16 (2,4)	**<0,001**	0,24 (0,17–0,31)	**<0,001**	0,08 (0,05–0,12)
≥0,15	19 (3,1)	7 (1,1)				
PI-RADS Score						
<4	613 (95,6)	2 (0,3)	**<0,001**	0,80 (0,77–0,84)	**<0,001**	0,78 (0,74–0,82)
≥4	5 (0,8)	21 (3,3)				

PSA, prostate-specific antigen; PI-RADS, Prostate Imaging Reporting and Data System.

*Univariate analysis; *p* value < 0.05 was considered for statistical significance (bold values within the table).

**Multivariable analysis; *p* value < 0.05 was considered for statistical significance (bold values within the table).

### Incidental extra-prostatic findings

Incidental extra-prostatic abnormalities were identified in 326/641 examinations (50.9%, 95% CI 47.0–54.8) (see Supplementary Table 2). The most common were colonic diverticulosis in 84/641 (13.1%, 95% CI 10.7–15.9) and inguinal hernias in 69/641 (10.8%, 95% CI 8.6–13.4), with bilateral cases in 1.4% (95% CI 0.7–2.6). Musculoskeletal changes included discopathy or Modic changes in 11/641 (1.7%, 95% CI 1.0–3.1). Less frequent but clinically relevant findings included bladder wall thickening or trabeculation suggestive of chronic outlet obstruction (5.9%, 95% CI 4.3–8.0), bladder lesions (0.3%, 95% CI 0.1–1.1) and suspicious lymphadenopathy or soft-tissue abnormalities requiring further evaluation (0.3%, 95% CI 0.1–1.1).

## Discussion

This study provides novel insights into the MRI characteristics of the prostate in a relatively young and asymptomatic male population, a cohort that remains underrepresented in the current literature. The findings highlight characteristic morphological and signal differences compared to older individuals, with important implications for image interpretation, diagnostic accuracy, and the development of potential screening strategies.

Evaluation of the peripheral zone revealed that most participants exhibited heterogeneous low signal intensity on T2-weighted imaging, observed in 74.7% of cases, while 10.3% showed a predominantly high T2 signal indicative of preserved glandular architecture. This observation aligns with prior studies showing that the peripheral zone in younger patients tends to appear hypointense or isointense on T2-weighted images, unlike the typically hyperintense signal seen in older individuals [29–31]. The reduced signal intensity has been attributed to the lower water content and shorter T2 relaxation times associated with stromal predominance and decreased glandular density [29, 30]. On diffusion-weighted imaging (DWI), the peripheral zone was most frequently homogeneously hyperintense (59.4%), with corresponding homogeneous hypointensity on ADC maps (66.1%), reflecting globally reduced apparent diffusion coefficients. This concordant pattern between DWI and ADC map likely represents a physiologically dense glandular microstructure, consistent with the higher cellular and stromal composition typical of younger men. Consistent with histological observations, the prostate gland in young adult males contains a higher proportion of compact fibromuscular stroma and fewer glandular acini, which translates directly into imaging findings. Such age-related differences are physiological rather than pathological, and they may be due to higher androgenic activity, increased glandular cellularity, and compact stromal tissue. However, they can mimic or obscure inflammatory or neoplastic processes, potentially complicating interpretation in a screening setting [30–32]. Recognizing these normal variants is therefore crucial to avoid overdiagnosis or unnecessary biopsy in younger patients undergoing prostate MRI. Our findings also show that men aged ≥60 years more often exhibit typical age-related features, including a hyperintense peripheral zone on T2-weighted imaging and absence of diffusion restriction. Additionally, the transitional zone may appear more homogeneous, lacking the heterogeneous nodular pattern commonly associated with benign prostatic hyperplasia (BPH) in older men [33]. These baseline characteristics must be carefully considered when evaluating young patients, particularly in the context of early screening or incidental findings, to avoid overdiagnosis and misclassification.

Despite the younger age and absence of clinical suspicion in our cohort, a notable proportion of participants exhibited PI-RADS ≥ 3 lesions, with clinically significant prostate cancer (csPCa) confirmed in 46.1% of those who underwent targeted biopsy. This detection rate, although based on a small subset, suggests that MRI could play a valuable role even in low-risk populations, enabling the early identification of clinically significant disease. Moreover, consistent with previous evidence, the use of biparametric MRI (bpMRI), which omits contrast media maintains a high diagnostic performance while remaining safe, non-invasive, and cost-effective for large-scale or preliminary screening applications [12, 14, 16, 18, 34].

While our study adopted an inclusive MRI-first approach without pre-screening based on PSA levels, we found that the median PSA values were higher among men undergoing biopsy. Although our study was not designed to identify a PSA threshold, and no dedicated analysis was performed on this aspect, we hypothesize that a PSA value around 2.5–3.0 ng/mL could serve as a contributing factor for risk-stratification of asymptomatic men [35, 36]. This threshold requires dedicated validation in future prospective cohorts. In the multivariate analysis, PSA density emerged as a strong independent predictor of csPCa, with values ≥ 0.15 ng/mL/mL markedly increasing the likelihood of its detection (*p* < 0,001). This finding is consistent with other MRI-based diagnostic studies, including evidence demonstrating the useful role of PSA-Density in refining risk stratification in biopsy-naïve men with clinical suspicion of PCa [23, 37]. Moreover, PI-RADS score also retained a significant independent association with csPCa, confirming its robustness as a diagnostic tool even in a younger screening cohort (*p* < 0,001). Interestingly, PI-RADS threshold of 4 was independently associated with csPCa detection in our multivariate analysis. This finding suggests that PI-RADS 4 may represent an appropriate biopsy threshold in a screening setting, mirroring evidence already established for men with clinical suspicion of prostate cancer [23, 38]. To mitigate the small yet clinically relevant risk of missing csPCa within PI-RADS 3 lesions, PSA density may serve as an effective risk-stratifying tool, helping to identify which equivocal lesions warrant further diagnostic work-up, a strategy that has similarly proven valuable in patients evaluated for clinical suspicion of prostate cancer [38]. Notably, family history of prostate cancer remained statistically significant (*p* = 0.003) in the adjusted model, indicating that hereditary risk contributes meaningfully to csPCa detection beyond PSA density and imaging features.

Our findings align with the principles of risk-adjusted screening strategies such as those proposed by the PROBASE trial [39]. PROBASE demonstrated that a baseline PSA < 1.5 ng/mL at age 45 identifies a large subgroup (89%) at very low 5-year risk for prostate cancer, with only 0.13 PCa cases per 1000 person-years. In our cohort, the PSA distribution was comparable, with a median of 1.02 ng/mL, suggesting a similar low-risk profile. Although PROBASE used PSA alone, our imaging-based approach may further refine early risk stratification. These complementary results support the potential of integrating PSA-based thresholds and imaging biomarkers (such as PSA Density and PI-RADS) into optimized early screening pathways for younger asymptomatic men.

Beyond prostate evaluation, incidental extra-prostatic abnormalities were documented in a considerable proportion of examinations. The most frequent findings were colonic diverticulosis (13.1%), inguinal hernias (12.2%), and renal cysts (5.5%). Although these findings are often clinically silent, they may hold diagnostic or prognostic relevance, especially in asymptomatic individuals. More severe but rare abnormalities, such as bladder lesions (0.3%) and suspicious lymphadenopathy (0.3%), further underscore the comprehensive diagnostic value of prostate MRI beyond cancer detection [19, 40].

Overall, these results suggest that MRI not only offers high diagnostic value for prostate cancer detection but also contributes to the characterization of normal prostate morphology and identification of incidental findings in younger populations. Recognizing the baseline MRI features of the young prostate is essential for accurate interpretation, minimizing false positives, and guiding evidence-based screening protocols.

This study has several limitations. First, it was conducted in a single center and included exclusively active-duty military personnel, a relatively homogeneous and healthy population, which may limit to the broader male population. Moreover, MRI interpretation was performed only by experienced radiologist; even though this ensured consistency, it may represent another factor limiting generalizability. Another limitation of this study is the use of a composite reference standard, whereby only men who underwent biopsy had histopathological confirmation, while negative status in the remaining participants was inferred from stable MRI findings or non-increasing PSA levels. Although unavoidable in a single-round screening design, and consistent with methodologies used in prior early-detection studies, this approach may introduce partial verification bias and could underestimate the prevalence of the disease. Nevertheless, it reflects real-world clinical practice and represents the most feasible and ethically acceptable verification strategy for asymptomatic, low-risk populations. Indeed, the cross-sectional design precludes longitudinal assessment of lesion evolution or progression, and broader follow-up data would be valuable to confirm the stability of MRI findings in younger individuals.

## Conclusions

In this cohort of younger, contrast-free MRI depicted normal prostate morphology and identified clinically significant prostate cancer at an early stage. The predominant peripheral-zone appearance, heterogeneous T2 hypointensity with corresponding DWI/ADC changes, likely reflects normal age-related tissue characteristics and should be recognized to avoid overinterpretation. PSA density, PI-RADS score, and family history were independent predictors of clinically significant disease, highlighting their value in refining risk stratification. Overall, these findings support contrast-free biparametric MRI as a feasible and non-invasive tool for early prostate evaluation in younger men, with PSA density and family history serving as key adjuncts to enhance diagnostic precision.

## Supplementary information

Supplementary Table 1.

Supplementary Table 2.

## References

[1] Sung H, Ferlay J, Siegel RL, Laversanne M, Soerjomataram I, Jemal A, et al. Global Cancer Statistics 2020: GLOBOCAN Estimates of Incidence and Mortality Worldwide for 36 Cancers in 185 Countries. CA A Cancer J Clin. 2021;71:209–49. 10.3322/caac.21660 33538338

[2] Printz C. Prostate cancer mortality projections reach a new high: With prostate cancer deaths projected to rise to their highest level in 20 years, some experts worry that changes to screening guidelines made in 2012 could be a factor. Cancer. 2020;126:3893–4. 10.1002/cncr.33127 32776531

[3] Siegel RL, Miller KD, Wagle NS, Jemal A. Cancer statistics, 2023. CA A Cancer J Clin. 2023;73:17–48. 10.3322/caac.21763 36633525

[4] EAU Guidelines. Edn. presented at the EAU Annual Congress Madrid 2025. ISBN 978-94-92671-29-5.

[5] Denijs FB, Van Harten MJ, Meenderink JJL, Leenen RCA, Remmers S, Venderbos LDF, et al. Risk calculators for the detection of prostate cancer: a systematic review. Prostate Cancer Prostatic Dis. 2024;27:544–57. 10.1038/s41391-024-00852-w 38830997

[6] Van Poppel H, Hogenhout R, Albers P, Van Den Bergh RCN, Barentsz JO, Roobol MJ. Early Detection of Prostate Cancer in 2020 and Beyond: Facts and Recommendations for the European Union and the European Commission. Eur Urol. 2021;79:327–9. 10.1016/j.eururo.2020.12.010 33384200

[7] Falagario UG, Lantz A, Jambor I, Busetto GM, Bettocchi C, Finati M, et al. Diagnosis of prostate cancer with magnetic resonance imaging in men treated with 5-alpha-reductase inhibitors. World J Urol. 2023;41:2967–74. 10.1007/s00345-023-04634-2 PMC10632288 37787941

[8] Cipollari S, Pecoraro M, Forookhi A, Laschena L, Bicchetti M, Messina E, et al. Biparametric prostate MRI: impact of a deep learning-based software and of quantitative ADC values on the inter-reader agreement of experienced and inexperienced readers. Radio Med. 2022;127:1245–53. 10.1007/s11547-022-01555-9 PMC9587977 36114928

[9] Turkbey B, Rosenkrantz AB, Haider MA, Padhani AR, Villeirs G, Macura KJ, et al. Prostate Imaging Reporting and Data System Version 2.1: 2019 Update of Prostate Imaging Reporting and Data System Version 2. Eur Urol. 2019;76:340–51. 10.1016/j.eururo.2019.02.033 30898406

[10] Barrett T, Rajesh A, Rosenkrantz AB, Choyke PL, Turkbey B. PI-RADS version 2.1: one small step for prostate MRI. Clin Radiol. 2019;74:841–52. 10.1016/j.crad.2019.05.019 31239107

[11] Garcia-Becerra CA, Arias-Gallardo MI, Juarez-Garcia JE, Soltero-Molinar V, Rivera-Rocha MI, Parra-Camaño LF, et al. Head-to-head comparison of diagnostic test accuracy between biparametric and multiparametric MRI: an updated systematic review and bivariate meta-analysis. Prostate Cancer Prostatic Dis. 2025;28:993–1004. 10.1038/s41391-025-00999-0 40696165

[12] Ng ABCD, Asif A, Agarwal R, Panebianco V, Girometti R, Ghai S, et al. Biparametric vs. Multiparametric MRI for Prostate Cancer Diagnosis: The PRIME Diagnostic Clinical Trial. JAMA. 2025;334:1170–9. 10.1001/jama.2025.13722 PMC12423955 40928788

[13] Siddiqui MR, Li EV, Kumar SKSR, Busza A, Lin JS, Mahenthiran AK, et al. Optimizing detection of clinically significant prostate cancer through nomograms incorporating MRI, clinical features, and advanced serum biomarkers in biopsy naïve men. Prostate Cancer Prostatic Dis. 2023;26:588–95. 10.1038/s41391-023-00660-8 36973367

[14] Eldred-Evans D, Burak P, Connor MJ, Day E, Evans M, Fiorentino F, et al. Population-Based Prostate Cancer Screening With Magnetic Resonance Imaging or Ultrasonography: The IP1-PROSTAGRAM Study. JAMA Oncol. 2021;7:395. 10.1001/jamaoncol.2020.7456 PMC7879388 33570542

[15] Hugosson J, Månsson M, Wallström J, Axcrona U, Carlsson SV, Egevad L, et al. Prostate Cancer Screening with PSA and MRI Followed by Targeted Biopsy Only. N Engl J Med. 2022;387:2126–37. 10.1056/NEJMoa2209454 PMC9870590 36477032

[16] Nam R, Patel C, Milot L, Hird A, Wallis C, Macinnis P, et al. Prostate MRI versus PSA screening for prostate cancer detection (the MVP Study): a randomised clinical trial. BMJ Open. 2022;12:e059482. 10.1136/bmjopen-2021-059482 PMC9644313 36351725

[17] Auvinen A, Tammela TLJ, Mirtti T, Lilja H, Tolonen T, Kenttämies A, et al. Prostate Cancer Screening With PSA, Kallikrein Panel, and MRI: The ProScreen Randomized Trial. JAMA. 2024;331:1452. 10.1001/jama.2024.3841 PMC10999002 38581254

[18] Messina E, Borrelli A, Sciarra A, Laschena L, Antonini D, Shaholli D et al. Primary Noncontrast Magnetic Resonance Imaging for Prostate Cancer Screening: A Randomized Clinical Trial (PROSA). Eur Urol 2025; S030228382504847X. 10.1016/j.eururo.2025.11.024 41412906

[19] Ponsiglione A, Campo I, Sachs C, Sofia C, Álvarez-Hornia Pérez E, Ciabattoni R, et al. Extraprostatic incidental findings on prostate mpMRI: A pictorial review from the ESUR junior network. Eur J Radiol. 2023;166:110984. 10.1016/j.ejrad.2023.110984 37480649

[20] Trivedi J, Sutherland T, Page M. Incidental findings in and around the prostate on prostate MRI: a pictorial review. Insights Imaging 2021; 12. 10.1186/s13244-021-00979-7. PMC7973355 33738590

[21] Messina E, La Torre G, Pecoraro M, Pisciotti ML, Sciarra A, Poscia R, et al. Design of a magnetic resonance imaging-based screening program for early diagnosis of prostate cancer: preliminary results of a randomized controlled trial—Prostate Cancer Secondary Screening in Sapienza (PROSA). Eur Radio. 2023;34:204–13. 10.1007/s00330-023-10019-1 37561183

[22] Boesen L, Nørgaard N, Løgager V, Balslev I, Bisbjerg R, Thestrup K-C, et al. Assessment of the Diagnostic Accuracy of Biparametric Magnetic Resonance Imaging for Prostate Cancer in Biopsy-Naive Men: The Biparametric MRI for Detection of Prostate Cancer (BIDOC) Study. JAMA Netw Open. 2018;1:e180219. 10.1001/jamanetworkopen.2018.0219 PMC6324414 30646066

[23] Messina E, Pecoraro M, Laschena L, Bicchetti M, Proietti F, Ciardi A, et al. Low cancer yield in PI-RADS 3 upgraded to 4 by dynamic contrast-enhanced MRI: is it time to reconsider scoring categorization? Eur Radio. 2023;33:5828–39. 10.1007/s00330-023-09605-0 PMC10326099 37045981

[24] Zambon A, Nguyen T-A, Fourcade A, Segalen T, Saout K, Deruelle C, et al. Which protocol for prostate biopsies in patients with a positive MRI? Interest of systematic biopsies by sectors. Prostate Cancer Prostatic Dis. 2024;27:500–6. 10.1038/s41391-023-00770-3 38114598

[25] Del Monte M, Cipollari S, Del Giudice F, Pecoraro M, Bicchetti M, Messina E, et al. MRI-directed biopsy for primary detection of prostate cancer in a population of 223 men: MRI In-Bore vs MRI-transrectal ultrasound fusion-targeted techniques. BJR. 2022;95:20210528. 10.1259/bjr.20210528 PMC8978234 34609900

[26] De Rooij M, Israël B, Tummers M, Ahmed HU, Barrett T, Giganti F, et al. ESUR/ESUI consensus statements on multi-parametric MRI for the detection of clinically significant prostate cancer: quality requirements for image acquisition, interpretation and radiologists’ training. Eur Radio. 2020;30:5404–16. 10.1007/s00330-020-06929-z PMC7476997 32424596

[27] Cipollari S, Guarrasi V, Pecoraro M, Bicchetti M, Messina E, Farina L, et al. Convolutional Neural Networks for Automated Classification of Prostate Multiparametric Magnetic Resonance Imaging Based on Image Quality. Magn Reson Imaging. 2022;55:480–90. 10.1002/jmri.27879 PMC9291235 34374181

[28] De Rooij M, Allen C, Twilt JJ, Thijssen LCP, Asbach P, Barrett T, et al. PI-QUAL version 2: an update of a standardised scoring system for the assessment of image quality of prostate MRI. Eur Radio. 2024;34:7068–79. 10.1007/s00330-024-10795-4 PMC11519155 38787428

[29] Suguino RK, Mussi TC, Coelho FMA, Baroni RH. Prostate imaging features on magnetic resonance imaging of young patients. Einstein. 2022;20:eAO0024. 10.31744/einstein_journal/2022AO0024 PMC9744424 36477522

[30] Bura V, Caglic I, Snoj Z, Sushentsev N, Berghe AS, Priest AN, et al. MRI features of the normal prostatic peripheral zone: the relationship between age and signal heterogeneity on T2WI, DWI, and DCE sequences. Eur Radio. 2021;31:4908–17. 10.1007/s00330-020-07545-7 PMC8213603 33398421

[31] Turkbey B, Huang R, Vourganti S, Trivedi H, Bernardo M, Yan P, et al. Age-related changes in prostate zonal volumes as measured by high-resolution magnetic resonance imaging (MRI): a cross-sectional study in over 500 patients. BJU Int. 2012;110:1642–7. 10.1111/j.1464-410X.2012.11469.x PMC3816371 22973825

[32] Messina E, Borrelli A, Mezzapesa F, Laschena L, Lucciola S, Novelli S et al. Artificial intelligence-assisted reading of non-contrast prostate MRI: application and concordance with expert interpretation in a screening population within the PROSA trial. Eur Radiol 2026. 10.1007/s00330-025-12269-7. 41686238

[33] Żurowska A, Pęksa R, Bieńkowski M, Skrobisz K, Sowa M, Matuszewski M, et al. Prostate Cancer and Its Mimics—A Pictorial Review. Cancers. 2023;15:3682. 10.3390/cancers15143682 PMC10378330 37509343

[34] Pecoraro M, Messina E, Bicchetti M, Carnicelli G, Del Monte M, Iorio B et al. The future direction of imaging in prostate cancer: MRI with or without contrast injection. Andrology 2021. 10.1111/andr.13041. 33998173

[35] Van Poppel H, Hogenhout R, Albers P, Van Den Bergh RCN, Barentsz JO, Roobol MJ. A European Model for an Organised Risk-stratified Early Detection Programme for Prostate Cancer. Eur Urol Oncol. 2021;4:731–9. 10.1016/j.euo.2021.06.006 34364829

[36] Laschena L, Messina E, Flammia RS, Borrelli A, Novelli S, Messineo D, et al. What the urologist needs to know before radical prostatectomy: MRI effective support to pre-surgery planning. Radio med. 2024;129:1048–61. 10.1007/s11547-024-01831-w PMC11252184 38918291

[37] Beetz NL, Dräger F, Hamm CA, Shnayien S, Rudolph MM, Froböse K, et al. MRI-targeted biopsy cores from prostate index lesions: assessment and prediction of the number needed. Prostate Cancer Prostatic Dis. 2023;26:543–51. 10.1038/s41391-022-00599-2 PMC10449625 36209237

[38] Pellegrino F, Stabile A, Sorce G, Quarta L, Robesti D, Cannoletta D, et al. Added Value of Prostate-specific Antigen Density in Selecting Prostate Biopsy Candidates Among Men with Elevated Prostate-specific Antigen and PI-RADS ≥3 Lesions on Multiparametric Magnetic Resonance Imaging of the Prostate: A Systematic Assessment by PI-RADS Score. Eur Urol Focus. 2024;10:634–40. 10.1016/j.euf.2023.10.006 37865591

[39] Krilaviciute A, Kaaks R, Seibold P, De Vrieze M, Lakes J, Radtke JP, et al. Risk-adjusted Screening for Prostate Cancer—Defining the Low-risk Group by Data from the PROBASE Trial. Eur Urol. 2024;86:493–500. 10.1016/j.eururo.2024.04.030 38749854

[40] Messina E, Pecoraro M, Pisciotti ML, Giudice FD, Lucciola S, Bicchetti M, et al. Seeing is Believing: State of the Art Imaging of Bladder Cancer. Semin Radiat Oncol. 2023;33:12–20. 10.1016/j.semradonc.2022.10.002 36517189

