# Supplementary material for: Contrast-free MRI-first screening in young asymptomatic men: Prostate characteristics and cancer detection rates
Source: Prostate Cancer Prostatic Dis. 2026 May 13;29(4):846–53. doi: 10.1038/s41391-026-01116-5 (PMC13623644; doi:10.1038/s41391-026-01116-5)
Supplement: Supplementary file 1 [file 41391_2026_1116_MOESM1_ESM.docx]

**Contrast-free MRI-First Screening in Young Asymptomatic Men: Prostate Characteristics and Cancer Detection Rates**

**Supplementary Table 1.** Parameters of the MRI image acquisition protocol.

| **MRI SEQUENCE** | **ACQUISITION PARAMETERS** | |
| --- | --- | --- |
| **T2WI (FSE)** | TE (ms) | 134 |
|  | TR (ms) | 6000 |
|  | Slice thickness (mm) | 3 |
|  | FOV (cm) | 12–20 |
|  | Matrix size | 320x224 |
| **DWI (EPI)** | TE (ms) | 75 |
|  | TR (ms) | 4300 |
|  | Slice thickness (mm) | 3 |
|  | FOV (cm) | 16–22 |
|  | Matrix size | 90x90 |
|  | Low b-value (sec/mm²) | 100 |
|  | Intermediate b-value (sec/mm²) | 800–1000 |
|  | High b-value (sec/mm²) | 2000 |

*T2WI, T2-weighted images; DWI, diffusion-weighted imaging; TE, time to echo; TR, repetition time*
