# Supplementary material for: Contrast-free MRI-first screening in young asymptomatic men: Prostate characteristics and cancer detection rates
Source: Prostate Cancer Prostatic Dis. 2026 May 13;29(4):846–53. doi: 10.1038/s41391-026-01116-5 (PMC13623644; doi:10.1038/s41391-026-01116-5)
Supplement: Supplementary file 2 [file 41391_2026_1116_MOESM2_ESM.docx]

**Contrast-free MRI-First Screening in Young Asymptomatic Men: Prostate Characteristics and Cancer Detection Rates**

**Supplementary Table 2.** Incidental extra-prostatic findings identified on prostate biparametric MRI (bpMRI).

| **Finding** | **n** | **% (95% CI)** |
| --- | --- | --- |
| Colonic diverticulosis | 84 | 13.1 (10.7–15.9) |
| Inguinal hernia (total) | 69 | 10.8 (8.6–13.4) |
| Inguinal hernia (bilateral) | 9 | 1.4 (0.7–2.6) |
| Renal cysts | 35 | 5.5 (4.0–7.5) |
| Hydroceles | 31 | 4.8 (3.4–6.8) |
| Discopathy or Modic changes | 11 | 1.7 (1.0–3.0) |
| Anterolisthesis | 9 | 1.4 (0.7–2.6) |
| Vertebral hemangiomas | 12 | 1.9 (1.1–3.2) |
| Tarlov or radicular cysts | 7 | 1.1 (0.5–2.2) |
| Epididymal cysts | 7 | 1.1 (0.5–2.2) |
| Bladder wall thickening/trabeculation | 38 | 5.9 (4.3–8.0) |
| Bladder diverticula | 2 | 0.3 (0.1–1.1) |
| Tailgut cyst | 1 | 0.2 (0.0–0.9) |
| Ureteroceles | 2 | 0.3 (0.1–1.19 |
| Cowper’s gland cysts | 3 | 0.5 (0.2–1.4) |
| Bladder lesions | 2 | 0.3 (0.1–1.1) |
| Suspicious lymphadenopathy/soft-tissue findings | 2 | 0.3 (0.1–1.1) |
